# Interaction with Intestinal Epithelial Cells Promotes an Immunosuppressive Phenotype in *Lactobacillus casei*

**DOI:** 10.1371/journal.pone.0078420

**Published:** 2013-11-07

**Authors:** Minna Tiittanen, Joni Keto, Johanna Haiko, Jaana Mättö, Jukka Partanen, Kaarina Lähteenmäki

**Affiliations:** Advanced Therapies and Product Development, Finnish Red Cross Blood Service, Helsinki, Finland; Cincinnati Children’s Hospital Medical Center, University of Cincinnati College of Medicine, United States of America

## Abstract

Maintenance of the immunological tolerance and homeostasis in the gut is associated with the composition of the intestinal microbiota. We here report that cultivation of *Lactobacillus casei* ATCC 334 in the presence of human intestinal epithelial cells promotes functional changes in bacteria. In particular, the interaction enhanced the immunosuppressive phenotype of *L. casei* as demonstrated by the ability of *L. casei* to generate functional regulatory T cells (CD4+CD25+FoxP3+) and production of the anti-inflammatory cytokine interleukin-10 by human peripheral blood mononuclear cells. The results indicate microbe-host cross-talk that changes features of microbes, and suggest that *in vitro* simulation of epithelial cell interaction can reveal functional properties of gut microbes more accurately than conventional cultivation.

## Introduction

The commensal microbiota in the intestine helps us to maintain the barrier function of intestinal mucosa and educates the gut immune system to function properly [1], [2]. It is estimated that there are at least 1000 different bacterial species in the intestine. Epithelial cells and phagocytic cells in the intestinal mucosa can recognize pathogenic and commensal bacteria with their pattern recognition receptors such as toll-like receptors (TLR). Signaling between commensal bacteria and TLR appears necessary for maintaining intestinal homeostasis and keeping up tolerance towards harmless non-self molecules [3], [4]. Disturbance in tolerance towards gut microbiota can lead to severe inflammation in the intestinal mucosa, which is characteristic for immunological disorders affecting the gut such as inflammatory bowel disease (IBD) [5].

Regulatory T cells (Treg) are CD4-positive, FOXP3-positive lymphocytes with immunosuppressive properties and ability to induce immunological tolerance [6], [7]. Natural Treg derive from the thymus and are believed to protect us from autoimmunity. Treg are also induced in the periphery during immunological activation. Induction of immunosuppressive Treg from naive CD4-positive T cells is especially important in creating immunological tolerance in the gut, and it was recently suggested that both natural Treg and induced Treg are essential for the establishment of full tolerance *in vivo* [8]. Immunosuppressive effect of Treg is mediated by many mechanisms, one of which is production of the anti-inflammatory cytokine interleukin-10 (IL-10) [9]. IL-10, which counteracts effects of proinflammatory cytokines such as tumor necrosis factor alpha (TNFα), prevents colitis in mouse models of human IBD [5].

Increasing evidence indicates that commensal microbiota affects Treg induction in the gut [9], [10]. Germ-free mice have markedly reduced amounts of Treg in the colon, and particular feces-derived *Clostridium* species that belong to clusters that are common in the intestinal microbiota of healthy individuals were recently found to induce IL-10 producing Treg in germ-free mice [11], [12]. Further, mice colonized with a mixture of commensal clostridia were less susceptible to chemically induced colitis than uncolonized mice [12]. Together with *Clostridium* group bacteria, which belong to the phylum *Firmicutes*, the phylum *Bacteroidetes* dominates in human intestinal microbiota [13], [14]. Also species belonging to *Bacteroidetes* can promote tolerance in the gut, as *Bacteroides fragilis* was shown to induce Treg and IL-10 production and to prevent and cure experimental colitis in mice [15], [16]. Like commensal bacteria, ingested probiotic bacteria can mediate tolerogenic functions in the gut [17], [18]. Expression of genes related to immune tolerance was induced in the duodenal mucosa of healthy human volunteers after consumption of *Lactobacillus plantarum* WCFS1 [19], and VSL#3 probiotic mixture induced Treg in mucosa of patients with ulcerative colitis [20]. In a murine model of IBD, oral administration of a lysate derived from the probiotic *Lactobacillus casei* DN-114001 increased Treg in mesenteric lymph nodes and protected mice from colitis [21]. In general, various beneficial effects have been reported for bacteria of *L. casei* group, which is formed by the taxonomically highly similar species *L. casei* and *L. paracasei* as well as the closely related *L. rhamnosus* [22].

Infection of the gut with pathogenic bacteria is, in general, followed by a proinflammatory cytokine response, as exemplified in mouse models with *Salmonella enterica* and *Citrobacter rodentium* [23], [24]. In these models, treatment with probiotic *Lactobacillus* species during or after pathogen infection increased IL-10 and Treg in the colon, and promoted mouse survival [23], [24], [25]. Thus, commensal and probiotic bacterial species can suppress overt inflammatory response mediated by pathogens or other stimuli and this way promote intestinal homeostasis.

*In vitro* studies done in human cell cultures have shown that the nature of bacterium-induced immune response depends not only on bacterial species but is also a strain-specific feature [26]–[30]. Specific strains of *Lactobacillus* have been found to increase the number of Treg and production of IL-10 in human peripheral blood mononuclear cells (PBMC) *in vitro* [30], [31]. A challenge in defining efficacy of probiotic preparations is that the *in vitro* effects of bacteria do not always correlate with effects *in vivo* [26], [29]. In most *in vitro* studies the bacteria have been cultivated in medium conditions that support efficient proliferation. Such conditions can be highly different from the intestinal environment. Models that consider the pH, bile and enzymatic conditions have been developed for simulation of microbial growth in the intestine [32]. However, microbial properties after interaction with intestinal epithelial cells have remained poorly characterized. We here address this issue by studying how immunomodulatory properties of *Lactobacillus casei* are affected when the bacterium is cultivated in the presence of human epithelial cells.

## Materials and Methods

### Ethics Statement

Human peripheral blood mononuclear cells (PBMC) were isolated from buffy coats left over in processing of blood collected from healthy blood donors. Buffy coats were handled anonymously, and their use for this project was approved by the ethical committee of the Finnish Red Cross Blood Service according to Finnish law. Before blood donation, donors are informed that blood samples that are not required for patient treatment can be used anonymously for research work if permission from the Finnish Red Cross Blood Service is obtained.

### Isolation of Human PBMC

PBMC were isolated from buffy coats by Ficoll-Paque Plus (GE Healthcare, Uppsala, Sweden) density gradient centrifugation according to the manufacturer’s instructions. The cells were washed three times with phosphate buffered saline, pH 7.2 (PBS) and suspended to RPMI 1640 culture medium (Gibco, Grand Island, NY) supplemented with 5% heat-inactivated (56°C, 30 min) human AB serum (Valley Biomedical, Winchester, VA), 100 U/ml Penicillin and 100 µg/ml Streptomycin (both from Gibco).

### Cultivation of HT-29 Cells

Human HT-29 (ATCC HTB-38) intestinal epithelial cells were cultivated to semiconfluency and divided onto 48-well or 6-well plates (Nunc, Roskilde, Denmark) at a concentration of 10^6^ cells/ml in McCoy’s 5A medium (Gibco) supplemented with 10% heat-inactivated fetal bovine serum (FBS; Gibco). The cells were cultivated at +37°C, 5% CO_2_ atmosphere overnight, after which the medium was removed and the wells were washed twice with PBS, and fresh, serum-free McCoy’s 5A medium was added onto the wells.

### Bacterial Strains

*Lactobacillus casei* ATCC 334 (VTT E-96710), *L. rhamnosus* VTT E-96666 (GG), *L*. *rhamnosus* VTT E-96031, *L. brevis* VTT E-82152, *L. reuteri* VTT E-92142 and *L. acidophilus* VTT E-96276 were purchased from VTT strain collection (VTT, Espoo, Finland) and *L. casei* DSM 20011 (ATCC 393) from the German collection of microorganisms and cell cultures (DSMZ). *L. plantarum* 299v was isolated from a probiotic product (Qur, Institut Rosell). Intestinal bacterial isolates were available from previous studies [33]. The isolates had been isolated from fecal samples of adult volunteers with no diagnosed gastrointestinal diseases [34]. Sample collection, ethical permissions and bacterial identification is described in [33], [34].

### Proliferation of Bacteria in the Presence of HT-29 Cells

For enumeration of bacterial growth on epithelial cells, *L. casei* ATCC 334 was cultured anaerobically in MRS broth (medium formulated by deMan, Rogosa and Sharpe; Tammer-Tutkan maljat Oy, Tampere, Finland) at 37°C overnight, collected by centrifugation and washed twice with PBS. The bacteria were inoculated into 48-well plates containing HT-29 cells and serum-free McCoy’s 5A medium at a concentration of 10^5^ bacteria/ml. Bacteria were similarly inoculated into plain, serum-free McCoy’s 5A medium, into serum-free medium collected from HT-29 cells after 18 hour incubation, or into MRS broth. The plates were incubated at +37°C, 5% CO_2_ atmosphere. After 18 hour cultivation, 0.2% Triton-X-100 (Sigma Aldrich, St Louis, MO) was added into all wells to ensure that also bacteria potentially adherent on HT-29 cells could be enumerated. After 45 minute incubation, samples were taken from the wells and series of ten-fold dilutions were plated on MRS agar plates (Tammer-Tutkan maljat Oy) for viable counting of bacteria. For rapid screening of bacterial strains, bacteria were inoculated onto HT-29 cells or into plain medium in 96-well plates (Nunc). The plates were incubated at +37°C, 5% CO_2_ atmosphere, and bacterial growth was detected by measuring absorbance at 595 nm.

### Cultivation of *L. casei* with HT-29 Cells or in MRS Broth

For functional comparison of *L. casei* ATCC 334 cultivated on HT-29 cells and in MRS broth, bacteria were first cultured anaerobically in MRS broth at 37°C overnight, collected by centrifugation and washed twice with PBS. Bacteria were then inoculated into 6-well plates containing HT-29 cells and serum-free McCoy’s 5A medium at a concentration of 10^6^ bacteria/ml. The plates were incubated at +37°C, 5% CO_2_ atmosphere. The bacteria were similarly inoculated into MRS broth and cultivated anaerobically. After 24 hour cultivation, bacterial cells were collected by centrifugation and washed three times with PBS. The optical density of bacterial suspensions was adjusted to OD600 = 1,0. Microscopic counting verified that with *L. casei* ATCC 334, this corresponds to approximately 10^8^ bacteria/ml.

### Co-culture of PBMC with *L. casei*

*L. casei* collected either from HT-29 cells or from MRS broth were co-cultured with 1.5×10^6^ PBMC in 48-well plates (Nunc) in 1∶1 ratio. For control, PBMC were also cultured in the absence of bacteria. The final volume of RPMI 1640 culture medium, supplemented with 5% heat-inactivated human AB-serum, 100 IU/ml penicillin and 100 µg/ml streptomycin, was 1 ml per well. Viable counts showed that the antibiotics inhibited the growth of bacterial cells.

Cell culture supernatant samples were collected after 24 hours, 5 days and 7 days for measuring the levels of secreted cytokines. Supernatants were kept frozen at −70°C until the analysis. PBMC were collected after 5 day co-culture, washed once with PBS containing 0.3% human serum albumin (Finnish Red Cross Blood Service, Helsinki, Finland) and 0.1% NaN_3_, and stained immediately for flow cytometric analysis.

### Cytokine Determination

Concentrations of secreted TNFα and IL-10 in supernatants were determined from co-cultures of seven PBMC preparations. All antibodies, standards and other reagents were purchased from BD Pharmingen (San Jose, CA) and a standard cytokine sandwich ELISA protocol was applied according to the manufacturer’s instructions. Briefly, 96-well Maxi Sorp microtiter plates (Nunc) were coated with a monoclonal capture antibody (mouse anti-human TNFα or rat anti-human IL-10), and after blocking with 1% bovine serum albumin (Sigma-Aldrich) in PBS, the samples were incubated in the wells. The bound cytokines were detected by biotinylated mouse anti-human TNFα or rat anti-human IL-10 antibody, avidin-horseradish peroxidase conjugate and tetramethylbenzidine substrate. The color reactions were stopped by adding 1 M phosphoric acid. The intensities of the color reactions were measured at 450 nm with Multiscan RC microplate reader (Thermo Fischer Scientific, Waltham, MA) and Multicalc 2000 software (Perkin Elmer, Waltham, MA). Two-fold dilutions of purified recombinant human TNFα and IL-10 were used as standards.

Human FlowCytomix kit (eBioscience, San Diego, CA) was used as a multiplex kit to measure the concentration of 12 chosen cytokines in supernatants from co-cultures of PBMC from three blood donors. The cytokines analyzed were IFNγ, IL-1β, IL-2, IL-4, IL-5, IL-6, IL-8, IL-10, IL-12 (p70), TNFα, TNFβ and IL-17 A/F. The assay was performed according to the manufacturer’s instructions and software.

### Detection of Treg by Flow Cytometry

Cells were stained for flow cytometric analysis of Treg using FOXP3 Staining buffer set (eBioscience) according to the manufacturer’s instructions. FITC-labeled anti-human CD4 antibody, PE-labeled anti-human CD127 antibody, PerCP-Cy5.5 -labeled FoxP3 antibody (all from eBioscience) and AlexaFluor 647-labeled anti-human CD25 antibody (BioLegend, San Diego, CA) were used to stain human cells after co-culture of *L. casei* and PBMC. Appropriate isotype controls were used to verify background fluorescence. Treg were gated in two different ways: 1) CD4+CD25^high^FOXP3+ lymphocytes and 2) CD4+CD25^high^CD127^neg^ lymphocytes (CD127 negativity has been shown to increase the specificity in gating CD4+CD25^high^ Treg [3], [35]). FACSCalibur machinery and FACS Diva software v5.0.3 (both from Becton Dickinson) were used in flow cytometric analyses.

### Immunosuppression Assay of Bacteria-induced Treg

2.4×10^8^ PBMC isolated from one buffy coat were co-cultured for 5 days with *L. casei* ATCC 334 collected from HT-29 cells. CD4+CD25+CD127^neg^ lymphocyte population (enriched Treg) was isolated by using magnetic separation after Ficoll-Paque Plus density gradient centrifugation. First, CD127 positive cells were depleted by using mouse anti-human CD127 antibody (eBioscience) and anti-mouse IgG MicroBeads (Miltenyi Biotec, Gladbach, Germany). In the second step CD4 positive cells were isolated with EasySep human CD4 positive selection kit (StemCell Technologies, Vancouver, Canada) and in the third step CD25 positive cells were isolated with EasySep human CD25 positive selection kit (StemCell Technologies). The isolation procedure was performed according to the manufacturers’ instructions except the buffer used in all steps was 2% human AB serum (Valley Biomedical) in PBS without ethylenediaminetetra-acetic acid (EDTA) which interferes with T cell immunosuppression.

Autologous PBMC (stored frozen in liquid nitrogen for 5 days) were stained in RPMI 1640 full culture medium with 5(6)-carboxyfluorescein diacetate *N*-succinimidyl ester (CFSE; InVitrogen Molecular Probes, Eugene, OR) for 5 min at room temperature and used as responder cells in the assay. Responder cells and isolated CD4+CD25+CD127^neg^ cells were cultured in 1∶1 ratio (5×10^4^ of both cells) in triplicate wells in round-bottom 96-well plates (Nunc) for 4 days in 5% CO_2_ atmosphere, and anti-CD3 antibody (100 ng/ml; clone HIT3a, BioLegend) was used as a stimulus in T cell proliferation. T cell proliferation was monitored by detecting CFSE fluorescence in lymphocytes in flow cytometry.

### Statistical Analysis

**Figure 1:**
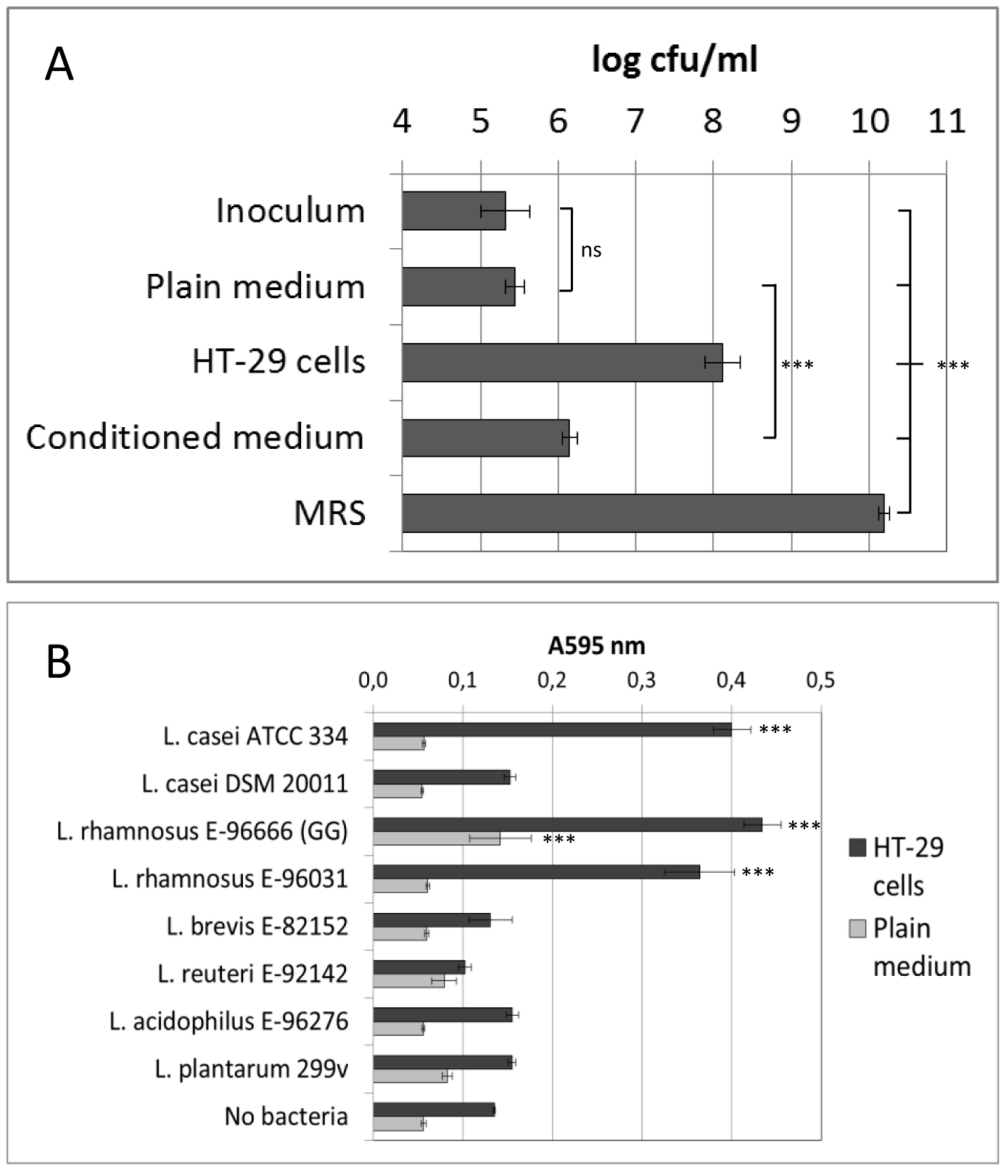
Effect of HT-29 intestinal epithelial cells on growth of *Lactobacillus* strains. A. *Lactobacillus casei* ATCC 334 was cultivated in MRS broth overnight, and then inoculated into McCoy’s 5A medium (plain medium), onto HT-29 cells in McCoy’s 5A medium (HT-29 cells), or into McCoy’s 5A medium that had been collected from HT-29 cells after 18 hour incubation (conditioned medium). Bacteria were also inoculated into MRS broth. After 18 hour cultivation, numbers of bacteria were determined by viable counting. Averages of three (conditioned medium), four (plain medium and MRS) or eight (HT-29 cells) replicate samples and standard deviations are shown. cfu = colony forming unit. ***, *p*<0.001; ns, non-significant (one-way Anova followed by Tukey’s Multiple Comparison test). B. *Lactobacillus* strains were inoculated onto HT-29 cells or into plain medium in 96-well plates. Bacterial growth was measured by absorbance at 595 nm. Averages of four replicate samples and standard deviations are shown. ***, samples differing from the corresponding non-bacteria control, *p*<0.001 (one-way Anova followed by Tukey’s Multiple Comparison test).

**Figure 2:**
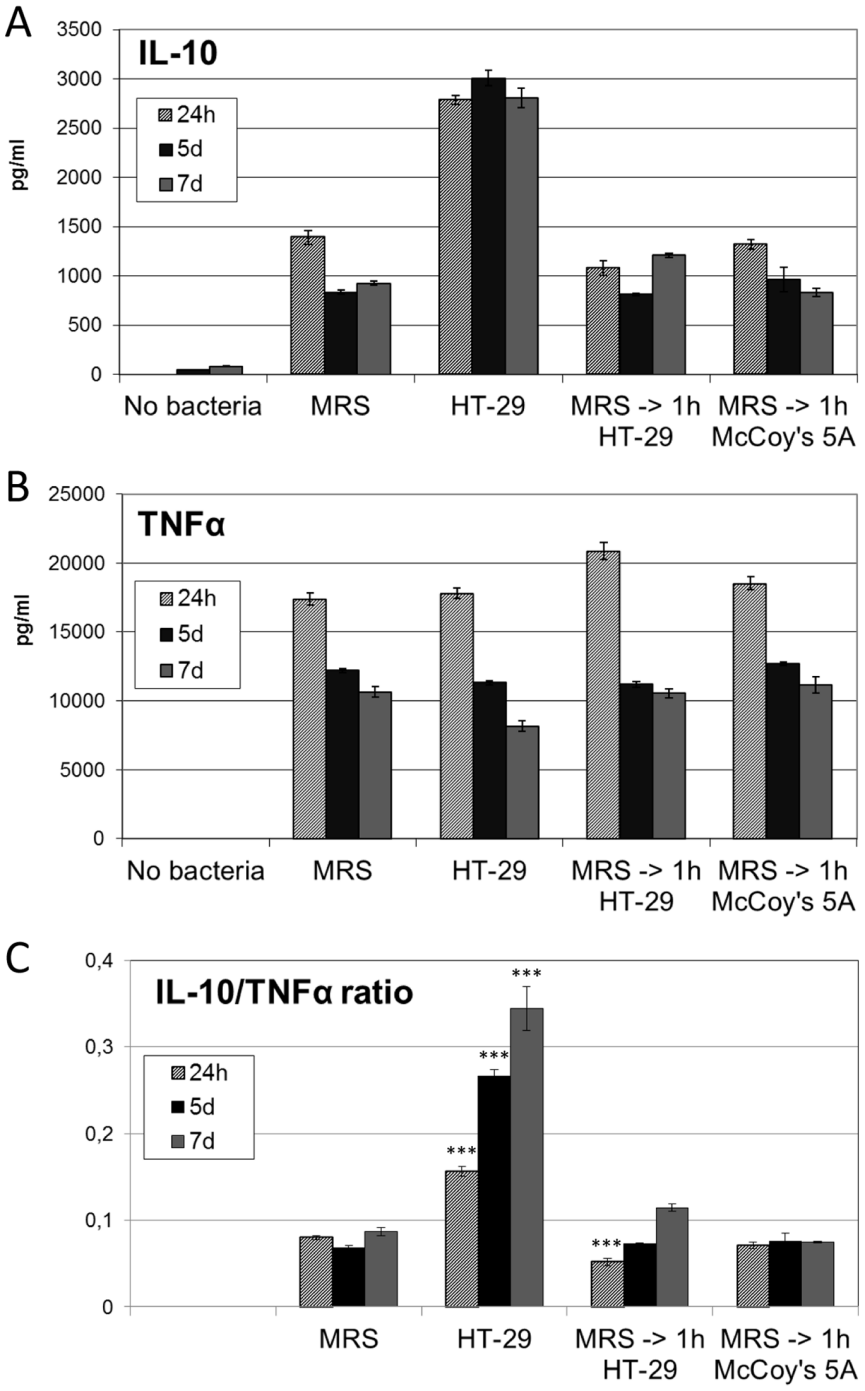
Effect of *Lactobacillus casei* ATCC 334 on IL-10 and TNFα production by PBMC. Bacteria were cultivated in the presence of HT-29 intestinal epithelial cells (HT-29) or in MRS broth (MRS), and then co-cultivated with PBMC isolated from one blood donor. After 24 hours, 5 days and 7 days, conditioned media from the cells were collected and amounts of interleukin-10 (IL-10; A) and tumor necrosis factor-alpha (TNFα; B) were determined by ELISA. Cytokine responses induced by bacteria cultivated in MRS broth and then incubated for 1 hour with HT-29 cells (MRS ->1 h HT-29) or in plain cell culture medium (MRS ->1 h McCoy’s 5A) are also shown. Averages of three technical replicates and standard deviations are shown. C. The ratio of IL-10/TNFα production by PBMC in the presence of bacteria is shown. ***, significant difference to IL-10/TNFα ratio induced by MRS-cultivated bacteria at the same time point (*p*<0.001; one-way ANOVA and Tukey’s Multiple Comparison test).

**Figure 3:**
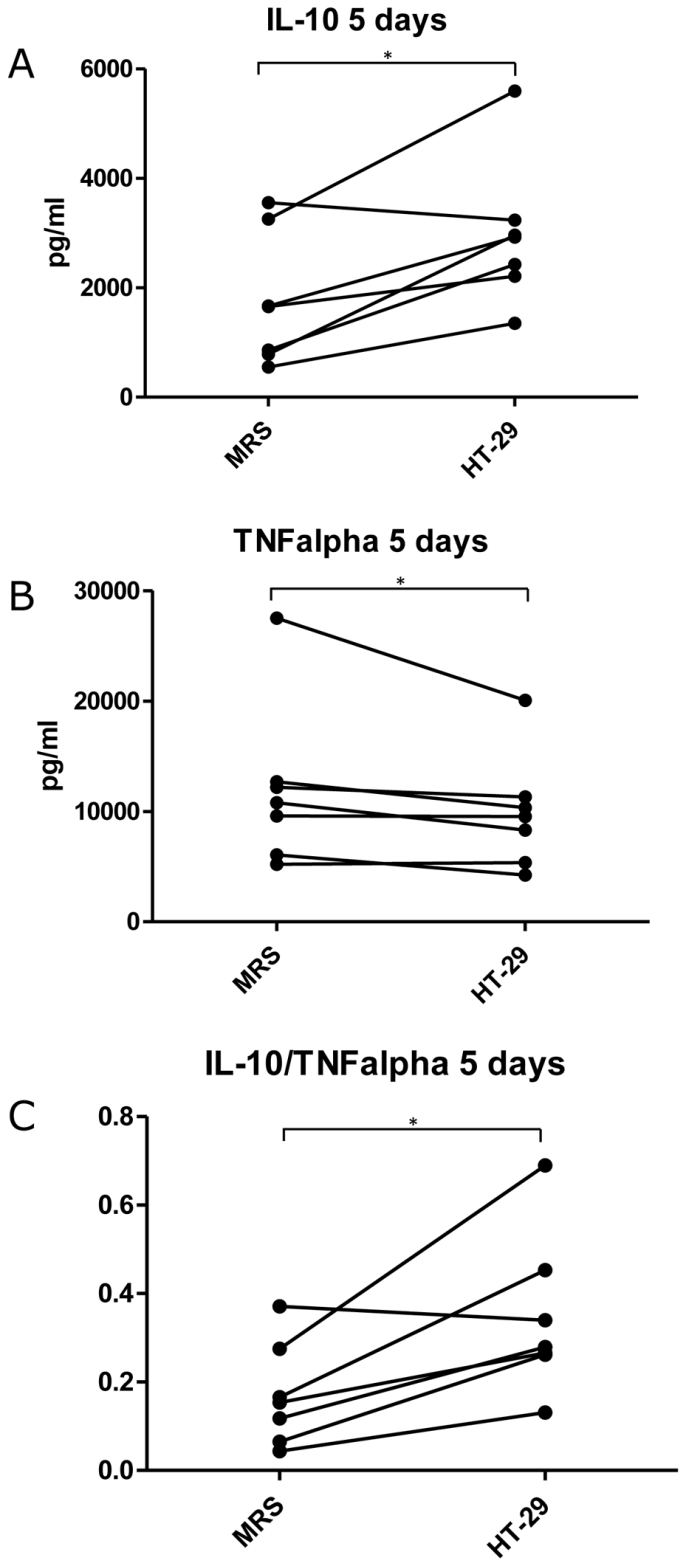
Effect of *Lactobacillus casei* ATCC 334 on IL-10 and TNFα production by PBMC from different individuals. Bacteria were cultivated in the presence of HT-29 intestinal epithelial cells (HT-29) or in MRS broth (MRS), and then co-cultivated with human PBMC (n = 7 donors). After 5 days, conditioned media from the cells were collected and the amounts of interleukin-10 (IL-10; A) and tumor necrosis factor-alpha (TNFα; B) were determined by ELISA. Averages of three technical replicates of each individual PBMC co-culture are shown. Background values (cytokine production in PBMC incubated in the absence of bacteria) were subtracted from the values measured in the presence of bacteria. The ratio of IL-10/TNFα is shown in graph C. *, *p*<0.05; Wilcoxon signed rank test.

**Figure 4:**
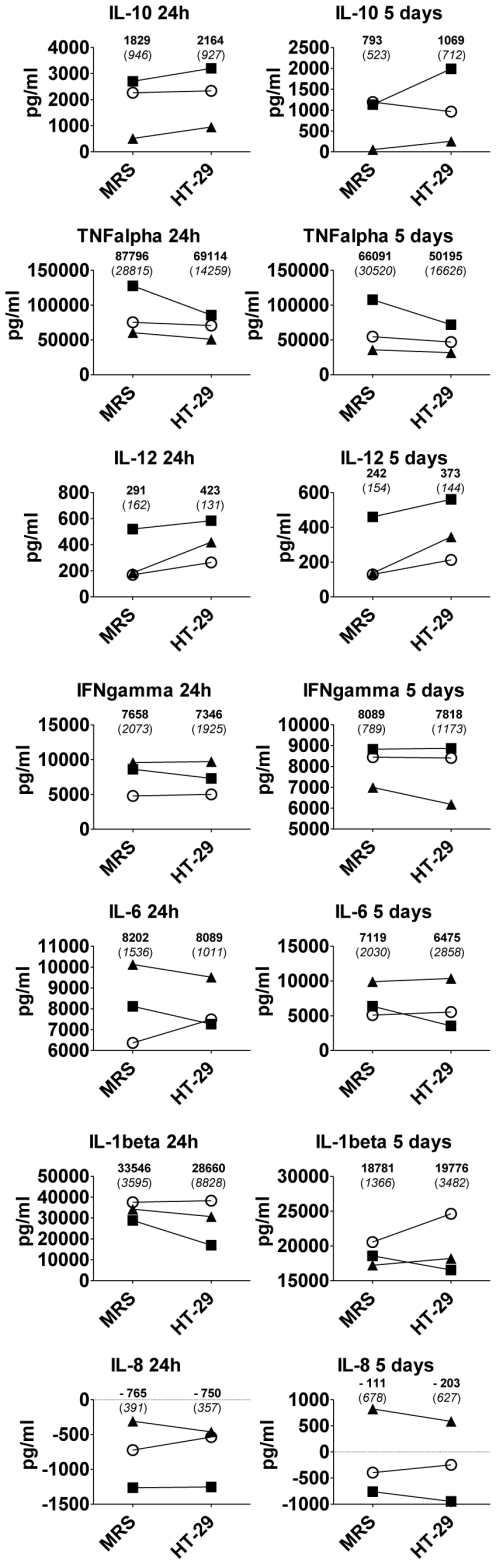
Effect of *Lactobacillus casei* ATCC 334 on production of a cytokine panel in PBMC. Bacteria were pre-cultivated in the presence of HT-29 intestinal epithelial cells or in MRS broth, and then co-cultured with human PBMC (n = 3 donors). After 24 hour and 5 day co-culture, conditioned media from the cells were collected and the amounts of indicated cytokines were determined by Human FlowCytomix kit. Background values (cytokine production in PBMC incubated in the absence of bacteria) were subtracted from the values. Cytokine production in each individual PBMC preparation is shown separately (squares, donor 1; circles, donor 2; triangles, donor 3). Averages (and standard deviations) of cytokine production in the three PBMC preparations are indicated. The data from 24 hour time-point originates from two technical replicates, and the data from 5 day time-point from one technical replicate of each PBMC.

**Figure 5:**
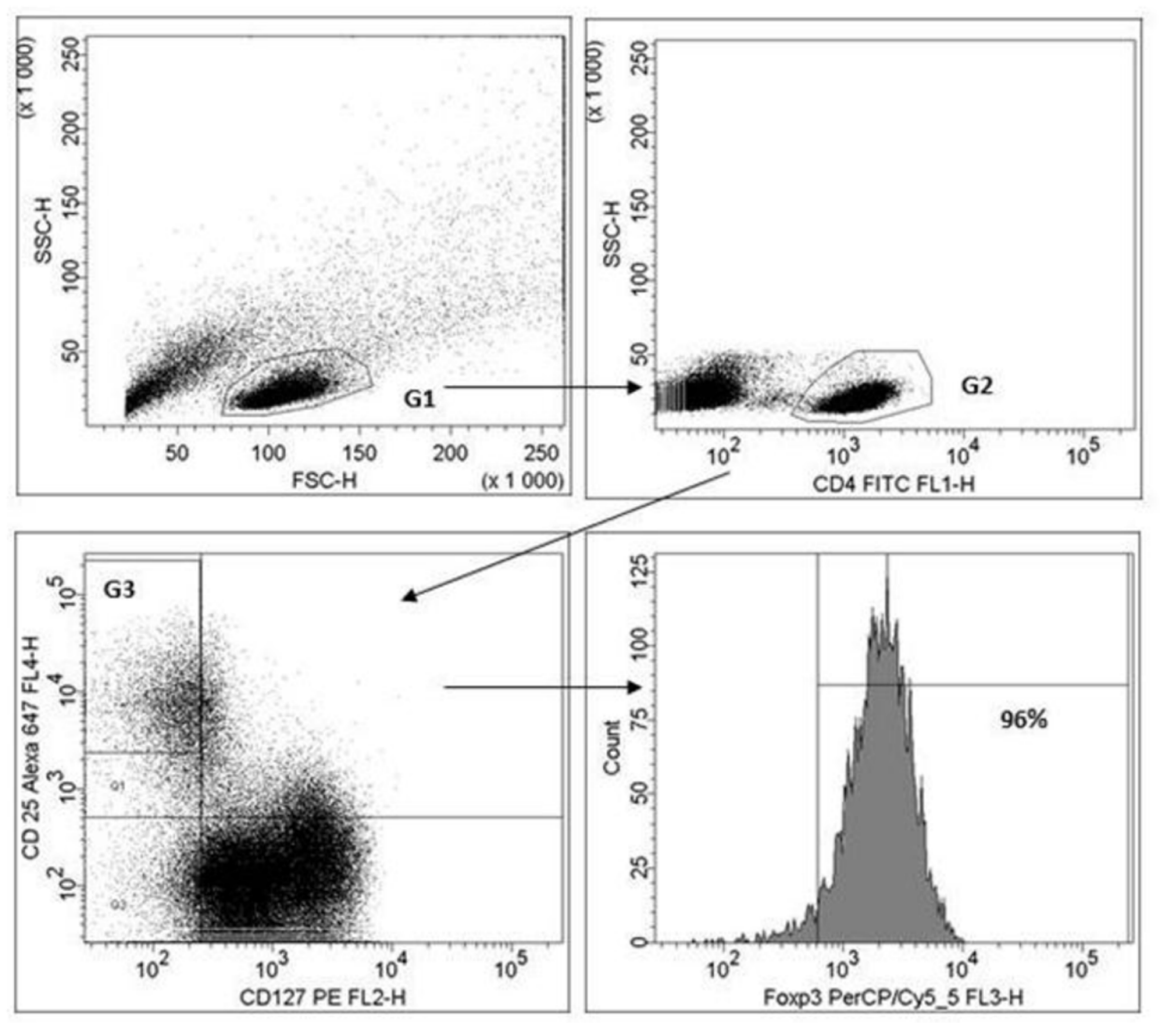
A representative example of gating CD4+CD25^high^CD127^neg^ lymphocytes in flow cytometry after 5 days co-culture of *Lactobacillus casei* ATCC 334 and PBMC from one blood donor. First lymphocytes ( = G1) are gated in FSC-SSC plot. From lymphocyte gate CD4-positive cells are gated in G2 from which the cells expressing high intensity CD25 and no CD127 are gated to represent regulatory T cells (G3). CD4+CD25^high^CD127^neg^ ( = G3) cells are shown to express FOXP3 in histogram in which the gate is placed according to isotype control.

**Figure 6:**
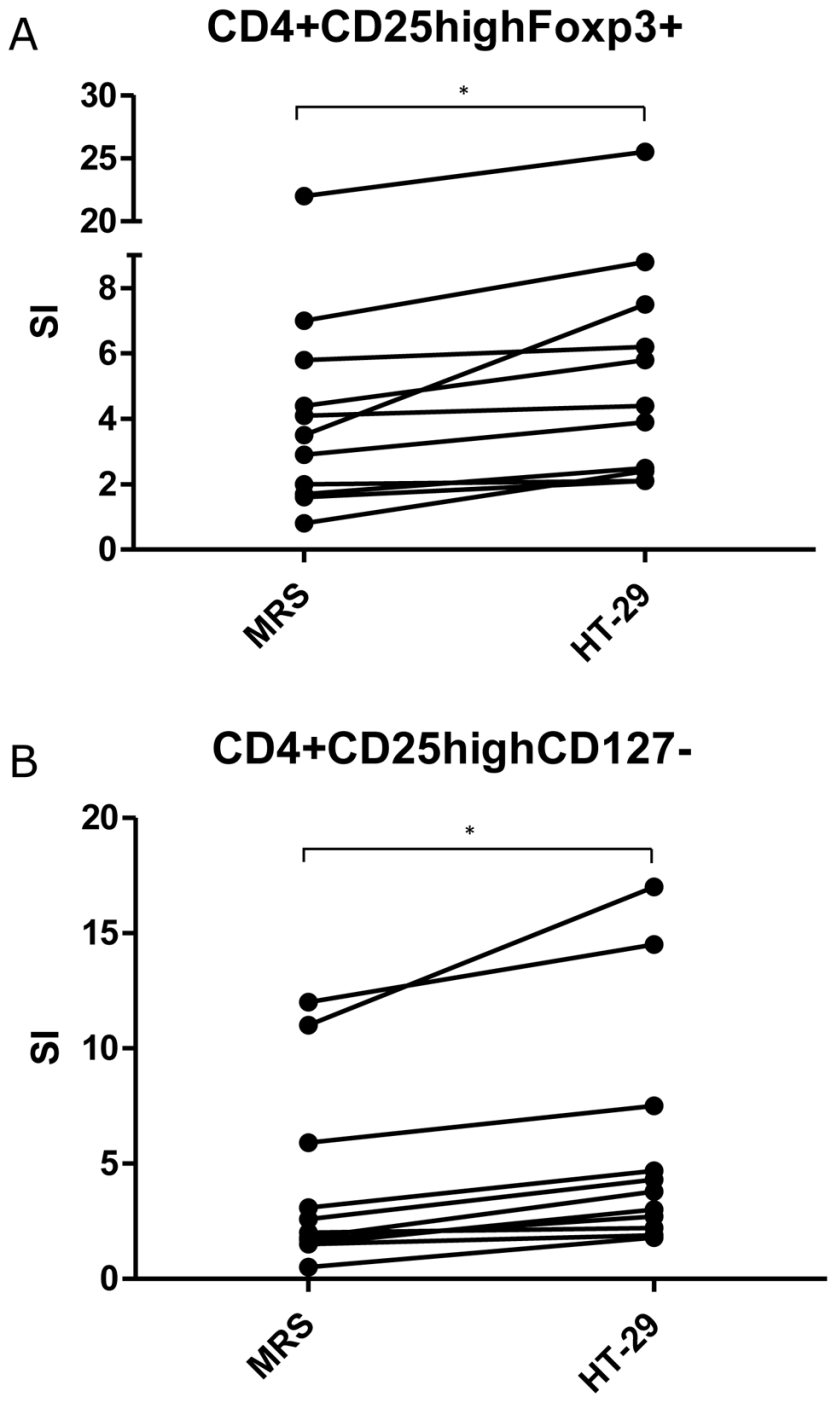
Regulatory T cells induced by *Lactobacillus casei* ATCC 334 in co-culture with PBMC. PBMC were co-cultured with bacteria pre-cultivated in MRS broth (MRS), with bacteria pre-cultivated on HT-29 epithelial cells (HT-29), or without bacteria (unstimulated control). After 5 day incubation, cells were stained for flow cytometry. Regulatory T cells were gated in two ways: CD4+CD25^high^FOXP3+ cells (A) or CD4+CD25^high^CD127^neg^ cells (B). The results are shown as stimulation indexes (SI) (percentage of regulatory T cells in PBMC stimulated with bacteria/percentage of regulatory T cells in PBMC in unstimulated control wells). *, *p*<0.05; Wilcoxon signed rank test.

**Figure 7:**
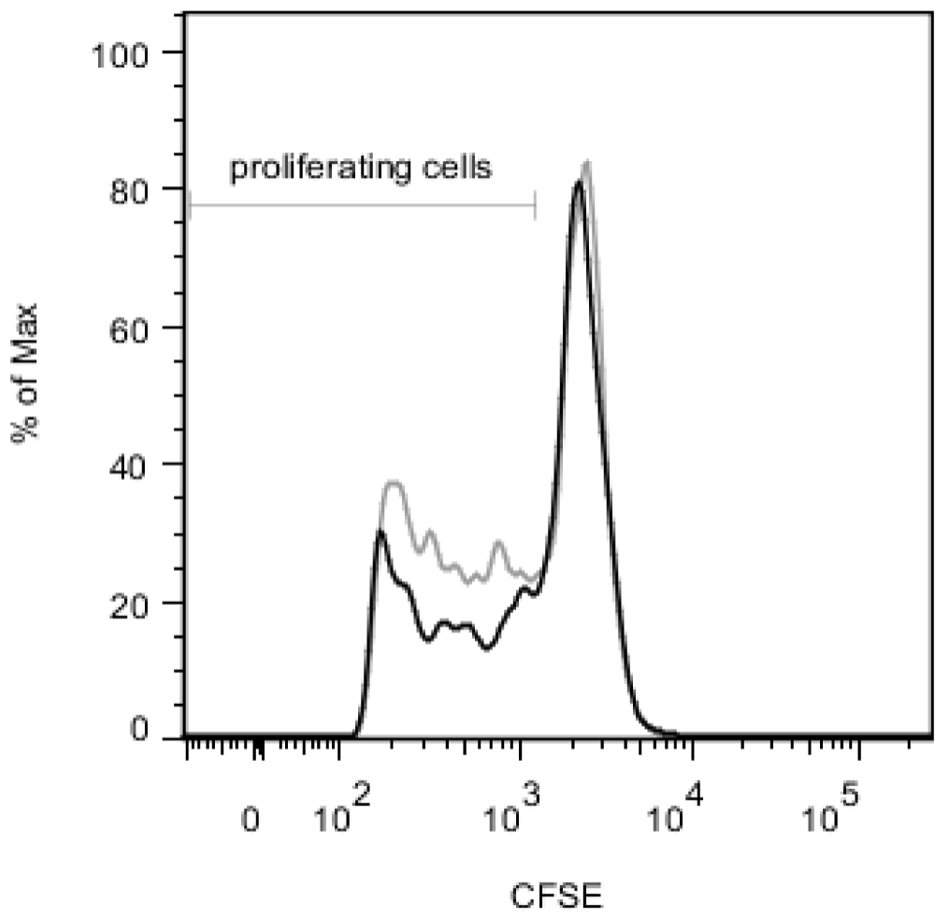
Immunosuppressive properties of regulatory T cells isolated from a co-culture of *Lactobacillus casei* ATCC 334 and PBMC. PBMC were co-cultured with bacteria pre-cultivated on HT-29 epithelial cells (HT-29). After 5 days, regulatory T cells were isolated using magnetic beads as CD4+CD25+CD127^neg^ cells. The proliferation of carboxyfluorescein diacetate *N*-succinimidyl ester (CFSE)-labeled autologous responder cells in a four-day T cell proliferation test without regulatory T cells is shown with grey line. Proliferation of the same responder cells with enriched regulatory T cells (in 1∶1 ratio) is shown with black line. Anti-human CD3 antibody was used as a stimulus in T cell proliferation test. A representative example of 3 replicate samples is shown. *p*<0.01 for the ratio of proliferating cells in 3 replicate samples with regulatory T cells vs 3 replicate samples without regulatory T cells (two-tailed unpaired t-test).

Statistical calculations were done by GraphPad Prism 5.04 software (GraphPad Software, San Diego, CA). The data sets in Figure 1 were analysed by one-way ANOVA and subsequent Tukey’s Multiple Comparison Test (Tukey). IL-10/TNFα ratios shown in Figure 2 were compared, at each time point separately, by one-way ANOVA and Tukey. In the data sets shown in Figure 3, effect of bacterial cultivation on PBMC response was analyzed by the non-parametric Wilcoxon signed-rank test. The cytokine data shown in Figure 4 was analyzed by paired t-test. Correlation between two gating strategies shown in Figure 5 was calculated by Spearman rank correlation test. The data sets in Figure 6 were analyzed as described for data sets in Figure 3. Spearman rank correlation test was used for calculating correlation between PBMC cytokine response and Treg production. For the data shown in Figure 7, unpaired t-test was used to compare responder cell proliferation in the presence and absence of Treg derived from bacterial co-culture. *P*-values <0.05 were considered significant.

## Results

### Intestinal Epithelial Cells Support Growth of *L. casei*

While developing cultivation conditions that would simulate the life of commensal organisms in host intestine, we observed that *L. casei* ATCC 334 could be cultivated on human intestinal epithelial cells. *L. casei*, which was inoculated into wells containing HT-29 cells in serum-free cell culture medium in approximately 10^5^ cfu/ml concentration, grew up to 10^8^ cfu/ml within overnight incubation (**Figure 1A**). Plain serum-free cell culture medium without the human cells did not promote proliferation of *L. casei* (*p*>0.05; plain medium vs inoculum; one-way ANOVA and Tukey; **Figure 1A**), indicating that the effect was due to epithelial cells (*p*<0.001; HT-29 cells vs plain medium and HT-29 cells vs inoculum; one-way ANOVA and Tukey). In the presence of conditioned serum-free medium collected from HT-29 cells a moderate growth of *L. casei* was noted, however, bacterial growth was considerably more efficient when epithelial cells were present. Growth on epithelial cells was not as efficient as in optimal cultivation medium, as in the standard laboratory culture medium MRS broth similar bacterial inocula grew to approximately 100-fold larger numbers (**Figure 1A**). However, although bacterial growth on epithelial cells was less efficient than in MRS broth, several population doublings took place during the co-culture of bacteria with HT-29 cells. Therefore, it is possible that the bacterial population that is derived from epithelial cell culture has phenotypic adaptations reflecting host cell interaction in the intestinal epithelium.

We next measured how HT-29 cells affect growth of certain commercially available *Lactobacillus* strains by measuring A595 after incubation of bacteria with HT-29 cells or in plain McCoy’s 5A medium. As shown in **Figure 1B**, in addition to *L. casei* ATCC 334, growth of *L.rhamnosus* E-96666 (GG) and *L. rhamnosus* E-96031 was also stimulated by epithelial cells (*p*<0.001 as compared to non-bacteria control; one-way ANOVA and Tukey). Growth of *L. casei* DSM 20011 (ATCC 393), L. *brevis* E-82152, *L. reuteri* E-92142, *L. plantarum* 299v and *L. acidophilus* VTT E-96276 on the cells was poor (*p*>0.05 as compared to non-bacteria control; one-way ANOVA and Tukey). *L.rhamnosus* GG was the only strain that also grew moderately in the presence of plain medium (*p*<0.001 as compared to non-bacteria control and to all other strains; one-way ANOVA and Tukey). A595 values measured for *L. casei* ATCC 334, *L.rhamnosus* GG and *L. rhamnosus* E-96031 on HT-29 cells corresponded to approximately 10^8^ cfu/ml in viable counting of the bacteria, indicating nearly 1000-fold increases in bacterial numbers from the original inoculum. A595 values measured for *L.rhamnosus* GG in plain medium corresponded to over 10^6^ cfu/ml, which indicates a 10-fold growth. Less efficient growth was not detectable by A595 measurement. Thus, A595 measurement is a robust method that is suitable for detection of large-scale differences between the strains, and we used it for prescreening of intestinal bacterial isolates for epithelial cell mediated growth effects. Nine of the 38 intestinal isolates studied showed similar growth characteristics as *L. casei* ATCC 334 (A595>0.3 with HT-29 and A595<0.06 in plain medium). Seven of these isolates belonged to *L. casei* group. Our results suggest that epithelial cell-stimulated proliferation is typical for bacteria of *L. casei* group, although strain-specific differences within the group exist. As *L. casei* ATCC 334 represented a strain with efficient proliferation on epithelial cells and virtually no growth in cell culture medium, we chose it for functional characterization after host cell-mediated growth.

### *L. casei* Cultivated in the Presence of Epithelial Cells Modulates PBMC Cytokine Secretion

To study the effect of epithelial cell cultivation on immunomodulatory functions of *L. casei*, we measured bacterium-induced cytokine production by human PBMC. *L. casei* was first cultured in the presence of HT-29 cells or in MRS broth. After collection and washing, the bacteria were co-cultured with PBMC in cell culture medium that contained antibiotics to prevent bacterial growth. Cell culture supernatants were collected after 24 hours, 5 days and 7 days, and the concentrations of TNFα and IL-10 were measured. PBMC incubated in the absence of bacterial cells produced negligible amounts of the cytokines, whereas bacteria from both cultivation conditions induced secretion of IL-10 and TNFα from PBMC (**Figure 2**). Notably, *L. casei* cultivated with HT-29 cells promoted significantly more secretion of IL-10 than bacteria cultivated in MRS broth (**Figure 2A**). Bacteria that were cultivated in MRS broth and then incubated only briefly (for 1 hour) on HT-29 cells or in plain cell culture medium induced an essentially similar amount of IL-10 as bacteria directly from MRS broth (**Figure 2A**). This indicates that the induction of high IL-10 production in PBMC required growth of *L. casei* on epithelial cells and could not be explained by epithelial cell-derived material attached on bacterial surface.

During 24 hour co-culture, epithelial-cell cultivated *L. casei* promoted a similar TNFα response in PBMC as bacteria from MRS broth, and after 5 and 7 day incubations, TNFα secretion was even slightly lowered (**Figure 2B**). This indicates that epithelial cell-cultivated bacteria did not induce a general increase in cytokine response. The ratio of IL-10 to TNFα was significantly higher in PBMC co-cultured with *L. casei* from epithelial cells already after 24 hours, and it further increased during longer incubation (*p*<0.001 at all time points; HT-29 vs MRS; one-way ANOVA and Tukey; **Figure 2C**). Similar difference to bacteria from control cultivations was also noted at all time points (*p*<0.001; HT-29 vs MRS ->1 h HT-29 and HT-29 vs MRS ->1 h McCoy’s 5A; one-way ANOVA and Tukey). On the contrary, a significant difference in IL-10/TNFα ratio between bacteria from control cultivations and MRS was noted only at 24 hour time point (*p*<0.001; MRS vs MRS ->1 h HT-29; one-way ANOVA and Tukey), but even then with a diminished ratio (**Figure 2C**). Altogether, the results suggest that epithelial cell cultivation turns the cytokine response mediated by *L. casei* ATCC334 to anti-inflammatory direction.

We verified the anti-inflammatory cytokine profile in PBMC obtained from 7 individuals. In all PBMC incubated in the absence of bacteria the concentrations of both IL-10 and TNFα were below the detection limit (data not shown). After 5 day co-culture with bacteria, production of IL-10 (**Figure 3A**) and TNFα (**Figure 3B**) increased in all PBMC, with a varying individual range. Notably, *L. casei* from HT-29 cells induced a higher level of IL-10 (*P* = 0.031 in Wilcoxon signed-rank test) and a lower level of TNFα (*P* = 0.047 in Wilcoxon signed-rank test) secretion (**Figure 3A–B**), and thus a significantly increased IL-10/TNFα ratio (*P* = 0.031 in Wilcoxon signed-rank test) as compared to *L. casei* from MRS broth (**Figure 3C**). Thus, despite of remarkable inter-individual differences in cytokine secretion, epithelial cell-derived bacteria consistently promoted a more anti-inflammatory cytokine response in PBMC.

To obtain a broader cytokine profile, we measured secretion of 12 cytokines from co-cultures of PBMC and *L. casei* using a flow cytometry-based multiplex kit. We used the same supernatants as in cytokine ELISA assays, and chose three PBMC that represented different types of donor responses to bacteria, including one with a high IL-10 response, one with a low IL-10 response, as well as the only PBMC where the detected IL-10 response was not stronger after co-culture with HT-29 cell-derived bacteria. With all cytokines that were secreted in detectable amounts, donor-dependent differences in the levels after bacterial stimulation were noted (**Figure 4** **)**. On the average, increased secretion of IL-10 and decreased secretion of TNFα in response to bacteria from HT-29 cells was noted also with this small subset of samples (**Figure 4** **)**, although no statistical significance could be seen. At 5 day samples, IL-10 was increased in two PBMC with bacteria from HT-29 cells and with one PBMC with MRS-cultivated bacteria, corresponding to the ELISA measurement. Despite of this, the average IL-10/TNFα ratio increased with bacteria from HT-29 cells at both time points. There also was a trend (*p* = 0.131, 24 hours; *p* = 0.079, 5 days; paired t-test) to higher secretion of IL-12 with bacteria from HT-29 cells (**Figure 4** **).** *L. casei*-induced response of the proinflammatory cytokines IFNγ, IL-6 and IL-1β varied at different time points and, in general, seemed not to be cultivation-dependent. As compared to PBMC incubated in the absence of bacteria, *L. casei* reduced secretion of the inflammatory chemokine IL-8, with an essentially similar efficiency from both culture conditions. IL-2, IL-4, IL-5, TNFβ and IL-17 A/F were not secreted in measurable amounts either in the presence or absence of bacteria (data not shown). The results, although done with a small subset of samples, support the view that epithelial cell cultivation does not cause a general increase in the cytokine response induced by *L. casei* ATCC 334. Instead, cultivation-dependent effects target mainly on increase in production of IL-10 and IL-12 by PBMC.

### *L. casei* Cultivated in the Presence of Epithelial Cells Induces Treg in PBMC Population

To study further how epithelial cell cultivation affects immunomodulatory properties of *L. casei*, we analyzed the number of induced Treg from co-cultures of bacteria and PBMC obtained from 11 different individuals. Treg were gated in two ways in flow cytometry: 1) percentage of CD4+ CD25^high^FOXP3+ lymphocytes, and 2) percentage of CD4+CD25^high^CD127^neg^ lymphocytes. An example of gating CD4+CD25^high^CD127^neg^ lymphocytes from one representative PBMC preparation when using *L. casei* from HT-29 cells is shown in **Figure 5**. The results were very similar between the two gating strategies (correlation coefficient 0.84 and *P*-value <0.0001 in Spearman rank correlation). The mean proportion of FOXP3-positive cells in CD4+CD25^high^CD127^neg^ lymphocyte gate was 85% (n = 11).

After 5 days co-culture, *L. casei* from both MRS broth and HT-29 cells clearly stimulated an increase in the number of Treg in PBMC, as compared to PBMC cultivated for 5 days in the absence of bacteria (unstimulated control) (**Figure 6**). When PBMC were co-cultured with *L. casei* from HT-29 cells the number of induced Treg, both CD4+ CD25^high^FOXP3+ (*P* = 0.001; Wilcoxon signed-rank test) and CD4+CD25^high^CD127^neg^ lymphocytes (*P* = 0.004; Wilcoxon signed-rank test), was higher than in co-culture with *L. casei* from MRS broth (**Figure 6**). The mean proportion of CD4+ cells of all lymphocytes after 5-day co-culture was 52% (SD 6.7%; n = 10; bacteria from HT-29), 50% (SD 5.6%; n = 10; bacteria from MRS), or 51% (SD 5.4%; n = 10; no bacteria). In one PBMC preparation that was studied in more detail, proportion of CD4+ cells was 43% before co-culture with bacteria, and increased to 47% (bacteria from HT-29), 48% (bacteria from MRS), or 49% (no bacteria) after 5-day co-cultivation. Thus, co-cultivation with epithelial cell derived bacteria seemed not to affect the proportion of the whole CD4+ population, but had a specific effect on Treg.

The results indicate that interaction with epithelial cells alters the functionality of *L. casei* so that it promotes efficient generation of Treg. Notably, the amount of induced Treg (CD4+CD25^high^CD127^neg^ lymphocytes) after 5 day co-culture correlated with the IL-10/TNFα ratio (correlation coefficient 0.62; *P* = 0.0174; Spearman rank correlation). Thus, the results suggest that *L. casei* cultivated with epithelial cells obtain properties that support an anti-inflammatory response by PBMC.

### Regulatory T cells Induced by Epithelial Cell-cultivated *L. casei* are Functional

We verified that the CD4+CD25+CD127^neg^ lymphocytes (enriched Treg) induced by *L. casei* collected from HT-29 cells were immunosuppressive by measuring their effect on T cell proliferation in a responder PBMC population. PBMC were isolated from a buffy coat derived from one donor and co-cultured with bacteria. *L. casei*-induced Treg cells were then enriched by CD127 depletion, followed by CD4 positive selection and CD25 positive selection. The cells enriched were co-cultured with CFSE-labeled autologous PBMC as responder cells in a T cell proliferation assay where T cell activation was induced by anti-CD3 antibody. After four days, on an average of 53% (range 52–56%, 3 technical replicates) of CFSE-labeled responder cells proliferated in response to anti-CD3 antibody (**Figure 7**). When the responder cells were co-cultured with *L. casei* –induced enriched Treg, the mean proportion of proliferating cells was only 42% (range 40–43%, 3 technical replicates; *p*<0.01; two-tailed unpaired t-test). The results indicate that the Treg population induced by *L. casei* cultivated with epithelial cells had immunosuppressive potential.

## Discussion

The present study shows that the interaction between bacterium and the host cell alters functional properties of the bacterium. We found that *L. casei* ATCC 334 can be cultivated *in vitro* with human intestinal epithelial cells as the sole nutrient source. Contact with human cells during the growth changed functional properties of *L. casei* so that it was more potent to increase the number of Treg and to promote an anti-inflammatory cytokine response by PBMC than after cultivation in the standard laboratory culture medium. It was recently published by López et al [36] that the presence of *Bifidobacterium bifidum* with intestinal epithelial HT-29 cells changed the gene expression profile of HT-29 cells. López et al also showed that supernatant from HT-29 cells previously treated with *B. bifidum* induced an increased amount of CD25^high^FOXP3+ Treg in PBMC culture. Thus, bacterium-host interaction induces changes to the host cells. Our results give evidence that this effect acts in both directions and show that immunosuppressive potential of *L. casei* was improved by growth with HT-29 cells.

Actual growth of bacteria with epithelial cells was required for the described functional changes, as bacteria that were first cultivated in MRS broth and then incubated only briefly with epithelial cells induced an essentially similar IL-10/TNFα ratio as bacteria directly from MRS broth. *L. casei* ATCC 334 efficiently proliferated with HT-29 cells, whereas its growth was poor in plain cell culture medium. We noted that also certain other *L. casei* group strains can proliferate similarly in the presence of epithelial cells. It is well known that proliferation of bacterial contaminants in cell cultures can be extremely efficient. In our previous study, we have shown that infection of host cell cultures with *Staphylococcus* and *Pseudomonas* species leads to at least five log increase in bacterial numbers within 24 hours [37]. On the contrary to pathogenic bacteria, proliferation of *L. casei* in cell culture conditions was moderate, and the number of bacteria remained at approximately 10^8^ cfu/ml even during prolonged incubation (data not shown). Also, on the contrary to the pathogenic contaminants, bacteria of *L. casei* group grew very poorly in plain cell culture medium. This indicates that the factors promoting *L. casei* growth are derived from epithelial cells.

An increase in PBMC IL-10/TNFα ratio in response to bacteria collected from epithelial cells was noted already after 24 hour co-culture, and the ratio further increased significantly in the 5-day co-culture. This was mainly due to reduced TNFα secretion rather than significantly increased IL-10 secretion. IL-10 most likely was derived from many cells types in the PBMC population, as in addition to Treg, various other cell types including monocytic cells and B cells produce IL-10 [38]. It is likely that after 24 hour incubation the secreted IL-10 derived predominantly from native cells present in PBMC, and at later time points also from Treg that had been induced from naïve CD4+ T cells. In line with our results, de Roock et al showed that in co-culture of PBMC and *Lactobacillus* strains, *Lactobacillus acidophilus* strain W55, which was the most efficient inducer of IL-10 production, also efficiently induced Treg in a 7-day co-culture [30].

IL-10 is a major anti-inflammatory cytokine, and probiotic strains stimulating IL-10 production are searched for their potential therapeutic effects in inflammatory disorders. Our results indicate that cultivation conditions affect the ability of *L. casei* ATCC 334 to induce IL-10 production. *L. casei* ATCC 334 is a widely used research strain, while specific strains of *L. casei*, *L. paracasei* and *L. rhamnosus* belong to most commonly used lactobacilli in probiotic products [39]. Thus, our results suggest that *in vitro* simulation of intestinal environment during selection of probiotic strains can be beneficial.

Our results also showed a trend towards higher IL-12 secretion after epithelial cell cultivation of bacteria. IL-12 is a pleiotropic cytokine that promotes T cell differentiation and NK cell cytotoxicity. The probiotic *L. casei* strain Shirota augments NK cell activity in mouse spleen after oral administration and increases production of IL-12 in mouse spleen cells *in vitro* [40]. *Lactobacillus*-induced IL-12 mediated NK cell activity is hypothesized to have beneficial effects in cancer treatment [41]. Interestingly, the pattern of IL-12 and IL-10 stimulation by *L. casei* Shirota in macrophages has been shown to be modulated by other, surrounding bacteria [41]. Thus, different types of cellular interactions in the intestinal environment can affect immunomodulatory functions in bacteria.

There clearly are significant individual variations in the immunological response of PBMC to microbial structures. Bacterial lipopolysaccharide and whole bacterial cells induce variable cytokine responses in PBMC, but the quantity and quality of the response is determined also by the individual responsiveness of PBMC [42], [43]. Our results show that also the level of Treg induction in response to bacteria varied greatly between cells from different individuals. It is of potential interest that the amount of Treg correlated well to the ratio of IL-10/TNFα, suggesting that individuals that react to bacterial stimulation with an immunosuppressive cytokine response also appear to produce higher levels of Treg. Despite of individual variation, *L. casei* cultivated with epithelial cells consistently induced a more anti-inflammatory response than bacteria from MRS broth.

According to the present study, cross-talk with host cells modifies immunomodulatory properties of gut bacteria in a way which may not be detected in standard laboratory environment. This cross-talk should be taken into consideration when studying properties of gut microbiota, and, in particular, when studying probiotic effects. Simulation of bacterial life in the intestinal environment by cultivation with intestinal epithelial cells can help in selection of therapeutic bacterial strains.
